# Aging Differentially Affects the Loss of Neuronal Dendritic Spine, Neuroinflammation and Memory Impairment at Rats after Surgery

**DOI:** 10.1371/journal.pone.0106837

**Published:** 2014-09-08

**Authors:** Yuan Le, Shuli Liu, Mingchao Peng, Chang Tan, Qin Liao, Kaiming Duan, Wen Ouyang, Jianbin Tong

**Affiliations:** 1 Department of Anesthesiology, the Third Xiangya Hospital, Central South University, Changsha, Hunan, P.R. China; 2 Center for Experimental Medicine, the Third Xiangya Hospital, Central South University, Changsha, Hunan, P.R. China; Massachusetts General Hospital, United States of America

## Abstract

It is known that age is an important factor for postoperative cognitive dysfunction (POCD) and the patients with POCD suffer from the impairment of multiple brain regions and multiple brain functions. However currently animal studies of POCD mainly focus on hippocampus region, therefore in this study we performed partial hepatectomy in young adult and aged rats to test the questions (1) whether POCD in animals involves other brain areas besides hippocampus; (2) how age influences POCD of young adult and aged animals. We found that (1) in young adult rats, the memory was not significantly affected (P>0.05) 1d, 3d and 7d after partial hepatectomy, but was significantly impaired (p<0.001) in aged rats 1d and 3d post-surgery; (2) in young adult rats, the surgery did not significantly affect the densities of dendritic spines of neurons at CA1, dentate gyrus (DG) and cingulate cortex (P>0.05, respectively) 1d and 3d post-surgery, but the spine densities at CA1 and DG of aged rats were significant reduced 1d and 3d post-surgery (p<0.001, respectively), however this didn’t happen at cingulate cortex (P>0.05); (3) In young adult rats, surgery didn’t affect the activation of microglia and levels of TNF-α and IL-1β at hippocampus (P>0.05), but significantly activated microglia and increased levels of TNF-α and IL-1β at hippocampus of aged rats (P<0.05). Our data suggest that (1) partial hepatectomy-induced POCD mainly involves hippocampus impairments, and (2) differential loss of neuronal dendritic spines and neuroinflammation at hippocampus are most likely the mechanism for the formation of POCD in aged rats.

## Introduction

Postoperative cognitive dysfunction (POCD) is usually detected among aged patients after surgery, especially after critical illness [1]–[3]. It characterizes with impaired memory, information processing, concentration and mental flexibility [1], [2], [4]. Occurrence of POCD is closely associated with increased incidence of postoperative complications, longer hospitalization, and higher mortality of 6 months [5]. The profound socioeconomic significance of POCD makes it the subject of many investigations.

Clinical researches have shown that brain areas involved in POCD include frontal, parietal, temporal, occipital, hippocampal, insular, cingulated, thalamic and cerebellar regions [4]. Risk factors of POCD include preoperative factors (such as age, cognitive function level, mental illness), perioperative factors (such as surgery type, duration of surgery, anesthesia type, intraoperative hypotension), and postoperative factors (such as postoperative infection, respiratory complications) [6]–[9]. Among these risk factors, age is the only risk factor for long-term POCD [6]–[9]. Further animal studies have shown that anaesthetics neurotoxicity, systemic inflammation induced by surgery trauma, and acceleration of ongoing endogenous neurodegenerative processes all contribute much to POCD [10]–[13]. For example, Terrando et al found that blocking the signals of TNF-α and IL-1 effectively decreased the impairment of cognitive function of adult mice after surgery [8]. Li et al found that minocycline, a drug of anti-inflammation, mitigated isoflurane-induced cognitive impairment in aged rats [14]. However, there is an obvious translational gap between clinical studies and animal studies of POCD [4]. Animal studies of POCD mainly focused on the changes of structures and functions of hippocampus induced by surgery, neglecting the fact of POCD that multiple brain regions and multiple brain functions are affected in patients. It remains unclear (1) whether POCD in animals involves other brain areas besides hippocampus; (2) how age influences POCD of young adult and aged animals.

The hippocampus and cingulate cortex are important structures involved in cognitive function [15]–[17]. Our previous study has shown that small volume of hippocampus is a valuable predictor of POCD in aged patients [2]. Hippocampal volume is negatively related with the score of neuropsychological tests in aged patients after surgery [2]. Cingulate cortex is associated with working memory, long-term memory, mental flexibility, and selective attention, which are totally impaired in POCD [4]. In addition, synapse plasticity is the structure base of cognitive functions and is usually evaluated by changes of dendritic spines of neurons [18]–[20]. For these reasons we answered above-mentioned two questions in this study by the detection of learning and memory, the measurement of the dendritic spine density of neurons at hippocampus and cingulate cortex, and the level of neuroinflammation of hippocampus at young adult and aged rats before and after surgery. We found that surgery induced obvious loss of dendritic spines of neurons at CA1 and dentate gyrus (DG) of aged rats, corresponding to their memory impairment after surgery. But surgery didn’t significantly affect dendritic spine density and memory of young adult rats. In addition, surgery also induced strong inflammation response at hippocampus of aged rats, but not at young adult rats. Our data suggest that postoperative memory dysfunction in aged rats is closely associated with the loss of neuronal dendritic spines and strong inflammation at hippocampus.

## Materials and Methods

### Animals and Grouping

The animal care and the experimental protocol were approved by the Institutional Review Board of the Third Xiangya Hospital of Central South University. Forty-six young adult (aged 2 months, 200–250 g) and fifty-two aged (aged 18 months, 500–610 g) female Sprague-Dawley rats were purchased from Central South University (P.R. China). All rats were raised under controlled environmental conditions on a 12 h light/dark cycle with ad libitum access to food and water.

Young adult (n = 22 for behavior test, n = 24 for Golgi staining and other staining) and aged rats (n = 28 for behavior test, n = 24 for Golgi staining and other staining) were randomly divided into normal control (n = 19 for young adult, n = 22 for aged), and surgery groups (n = 27 for young adult, n = 30 for aged). Rats in surgery group received partial hepatectomy plus general anethesia. Rats in nomal control received neither anesthesia nor surgery.

### Anesthesia and partial hepatectomy

Anesthesia was prepared with the procedure described by He et al [12]. Rats were exposed to sevoflurane (4.5% sevoflurane in 100% oxygen for induction followed by 2.5% for maintenance) for 2 h with endotracheal intubation with a 14-gauge catheter. Gas concentrations and respiratory rate were continuously monitored with a multi-function monitor (datex-ohmeda, Helsinki, Finland).

Partial hepatectomy was performed under aseptic conditions following Wuri’s method [21]. Briefly, a small incision about 2 cm was made in the upper quadrant through skin and muscles. The left liver was visualized, isolated, and removed. After checking, the muscles and skins were sutured, respectively.

### Morris Water maze test

The Morris water maze test was used for assessing learning and memory of rats. We used a computerized video track system (Logitech, Suzhou, China) to record the rat’s movement in water maze by following our previous method [12]. Briefly, a transparent round platform was placed below the water surface of southeast quadrant in a circular pool. During the training, rats were first placed on the platform for 30 seconds, and then were released into the water facing the tank wall. The maximum trial time was 60 seconds for a trial, following a relaxation of 15 seconds on the platform. If a rat couldn’t locate the platform within 60 seconds, it was guided to the platform and remained for 30 seconds. All rats were trained for 6 days with three trials per day. After training, the memory of rats, which was evaluated by the latency for the first entrance of targeted area and the percents of searching time and distance in the targeted area, was detected 1d before surgery and 1d, 3d, and 7d after surgery.

### Golgi staining and analysis

Golgi staining was performed with FD Rapid Golgi-stain Kit (FD Neurotechnologies, MD) according to the manufactures instructions [18]–[20]. Briefly, fresh brain was impregnated in the mixed solution A and B for two weeks at room temperature, and then at solution C for 48 hours at 4°C. The brains were cut with 150 µm thickness and mounted on the gelatin-coated slides. After drying, the sections were stained with solution D and E, dehydrated in graded ethanol, cleared in xylene, and finally covered by coverslip. Neurons (5 CA1 neurons/rat, 5 neurons at the 2^nd^ layer of cingulate cortex/rat, 8 DG neurons/rat) were analyzed following our previous methods [18]–[20]. The selected neurons have to be relatively isolated from neighboring neurons. Three to five tertiary apical dendrites and basal dendrites with at least one branch point were selected for counting for each neuron [18]–[20]. The visible spines along the branch segment (>10 µm long) were counted and data were expressed as number/10 µm [18]–[20].

### Tissue Preparation

For immunofluorescence assay, under deep anesthesia, rats were firstly infused with 0.9% saline at 37°C, and then with 4% paraformaldehyde. The brains were taken out, and then post-fixed in 4% paraformaldehyde overnight at 4°C. After dehydrating with sucrose, cross sections with hippocampus were cut at a thickness of 16 µm by a cryostat machine.

For western blot assay, under deep anesthesia, rats were killed. The hippocampus was removed and homogenized in cold lysis buffer containing protease inhibitors. After centrifuging, the supernatant of hippocampal homogenates were collected and stored at −80°C.

### Immunofluorescence

After washing with 0.01 M phosphate buffered saline (PBS) for 10 min, sections were incubated in blocking solution (5% BSA and 0.3% Triton X-100 in 0.01 M PBS) for 1 hr at room temperature. Then the sections were incubated in primary antibodies (rabbit anti-Iba-1, lot 019–19741, 1∶1000, Wako Chemical) overnight at 4°C. On the second day, these sections were washed with 0.01 M PBS for three times and then incubated in the secondary antibodies labeled with fluorescent dyes (1∶200, Jackson Immunoresearch) for 2 hours at room temperature. Through three washes of PBS, these sections were covered with mounting medium with DAPI (vector). As negative controls, an adjacent series of sections were processed using the same procedures without the primary antibodies.

### Western blot

The quantity of protein of samples was determined using a BCA protein assay kit (Wellbio, China) according to the manufacturer’s instructions. Equal amounts of protein samples (/lane) were separated by sodium dodecyl sulfate polyacrylamide gel electrophoresis (SDS-PAGE) and transferred to polyvinylidene fluoride membranes. Membranes were blocked with 10% skim milk in TBST buffer for 1 h and then incubated with primary antibodies (rabbit anti-TNF-α polyclonal antibody, 1∶1000, Abcam, Cambridge, UK; rabbit anti-IL-1β polyclonal antibody, 1∶1000, Abzoom, Shanghai, China;rabbit anti-GAPDH, 1∶4000, Proteintech Group, Shanghai, China) overnight at 4°C. After three washes, membranes were incubated with the secondary antibodies (1∶2000) at room temperature for 2 h. Finally, visualization of the proteins was accomplished by enhanced chemiluminescence detection kit (Pierce; Thermo Scientific, Shanghai, China), and the intensity of each band was quantified by densitometry. Relative expression levels of protein were normalized by the ratio of target protein (TNF-α, IL-1β) to GAPDH.

### Statistical analysis

Water maze data were presented as mean ± standard error (mean ± SEM). The water maze data were analyzed using repeated measures ANOVA with the factor surgery and the factor measure time, complemented by repeated measures ANOVA at data of control group and surgery group, respectively. Golgi data and western data were presented as mean ± standard deviation (mean ± SD) and were analyzed using two-way ANOVA followed by LSD test. P<0.05 was considered statistically significant.

## Results

### Hepatectomy differentially impaired the memory of young adult and aged rats

**Figure 1:**
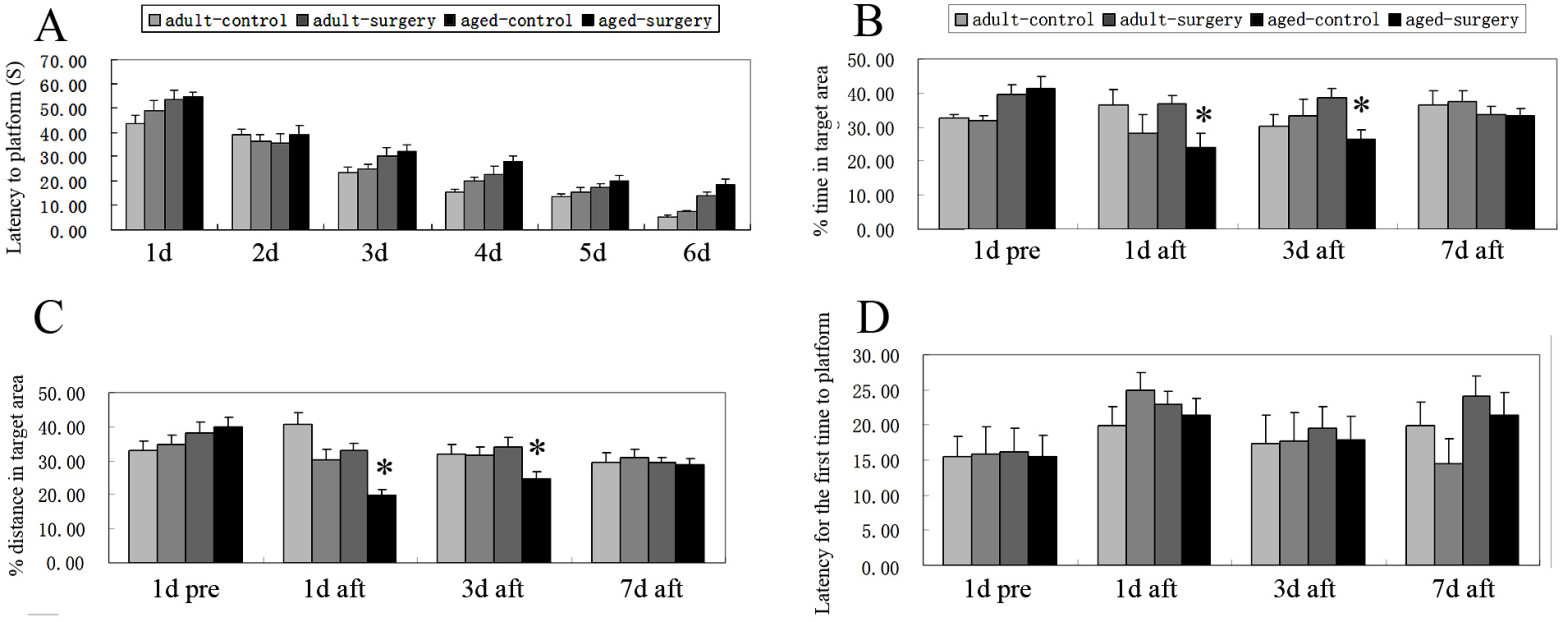
Hepatectomy differentially impaired the memory of young adult and aged rats. Latency to platform of young adult and aged rats both at control and surgery groups decreased with the increase of training days. No significant difference was detected between control and surgery groups of young adult and aged rats by repeated measures of ANOVA (P = 0.202 for aged; P = 0.057 for young adult) (A). During probe test of aged rats, repeated measures of ANOVA showed that the latency for the first entrance of targeted area wasn’t affected by surgery (P = 0.495), measure time (P = 0.067), and the interaction of surgery×measure time (P = 0.984) (D). Yet, percents of time (B) and distance (C) in the targeted area both were significantly affected by surgery (P = 0.045 for time; P = 0.041 for distance), measure time (P = 0.013 for time; P<0.001 for distance), and the interaction of surgery×measure time (P = 0.037 for time; P = 0.013 distance). Percents of time (B) and distance (C) in the targeted area of aged rats 1d (P = 0.006 for time; P<0.001 for distance) and 3d (P = 0.011 for time, P = 0.002 for distance) after surgery all were obviously less than that of 1d before surgery, respectively. For young adult rats, there was no obvious difference at the percent of distance in the targeted area (P = 0.170), the percent of time in the targeted area (P = 0.772) and the latency for the first entrance of targeted area (P = 0.938) between control and surgery groups 1d before surgery (A). Percents of time and distance, and the latency for the first entrance in the targeted area were not affected by surgery (P = 0.639 for time; P = 0.773 for distance; P = 0.983 for latency) and measure time (P = 0.327 for time; P = 0.358 for distance; P = 0.173 for latency) (B, C, D). 1d pre: 1d before surgery; 1d aft, 3d aft and 7d aft: 1d, 3d, 7d after surgery. Data were mean ± SEM. *p<0.05 *vs* 1d before surgery.

The effects of surgery on the memory of rats were detected by Morris water maze. During the training of 6 days, there was no significant difference of the latency to platform between control and surgery groups in aged (P = 0.202) and young adult rats (P = 0.057), suggesting same learning ability in control and surgery groups (Fig. 1A). During the probe test, the latency for the first entrance of targeted area and the percents of time and distance in the targeted area were measured to evaluate the memory of rats. Percent of distance in targeted area (P = 0.767), percent of time in targeted area (P = 0.769) and latency for the first entrance of targeted area (P = 0.903) in the surgery group weren’t different from that of control group at aged rats 1d before surgery (Fig. 1 B–D), suggesting that the same memory ability of aged rats at control group and surgery group before surgery. However, further repeated measures ANOVA showed that percents of searching time and distance in the targeted area of aged rats were affected by surgery (P = 0.045 for time; P = 0.041 for distance), measure time (P = 0.013 for time; P<0.001 for distance), and the interaction of surgery×measure time (P = 0.037 for time; P = 0.013 distance) (Fig. 1B, C). Compared to 1d before surgery, the percents of time and distance in the targeted area of aged rats obviously decreased 1d (P = 0.006 for time, P<0.001 for distance) and 3d (P = 0.011 for time, P = 0.002 for distance) after surgery (Fig. 1B, C). The latency for the first entrance of targeted area at aged rats was not affected by surgery (P = 0.495), measure time (P<0.067), the interaction of surgery×measure time (P = 0.984) (Fig. 1D). These above suggested that surgery impaired the memory of aged rats 1d and 3d after hepatectomy.

For young adult rats, there was no obvious difference at the percent of distance in the targeted area (P = 0.170), the percent of time in the targeted area (P = 0.772) and the latency for the first entrance of targeted area (P = 0.938) between control and surgery groups 1d before surgery (Fig. 1). Further repeated measures ANOVA showed that the percents of time and distance, and the latency for the first entrance in the targeted area were not affected by surgery (P = 0.639 for time; P = 0.773 for distance; P = 0.983 for latency), measure time (P = 0.327 for time; P = 0.358 for distance; P = 0.173 for latency) and the interaction of surgery×measure time (P = 0.122 for time; P = 0.095 for distance; P = 0.436 for latency) (Fig. 1). These data suggested that surgery didn’t impair the memory of young adult rats.

### Hepatectomy differentially induced the loss of dendritic spines of neurons at young adult and aged rats

**Figure 2:**
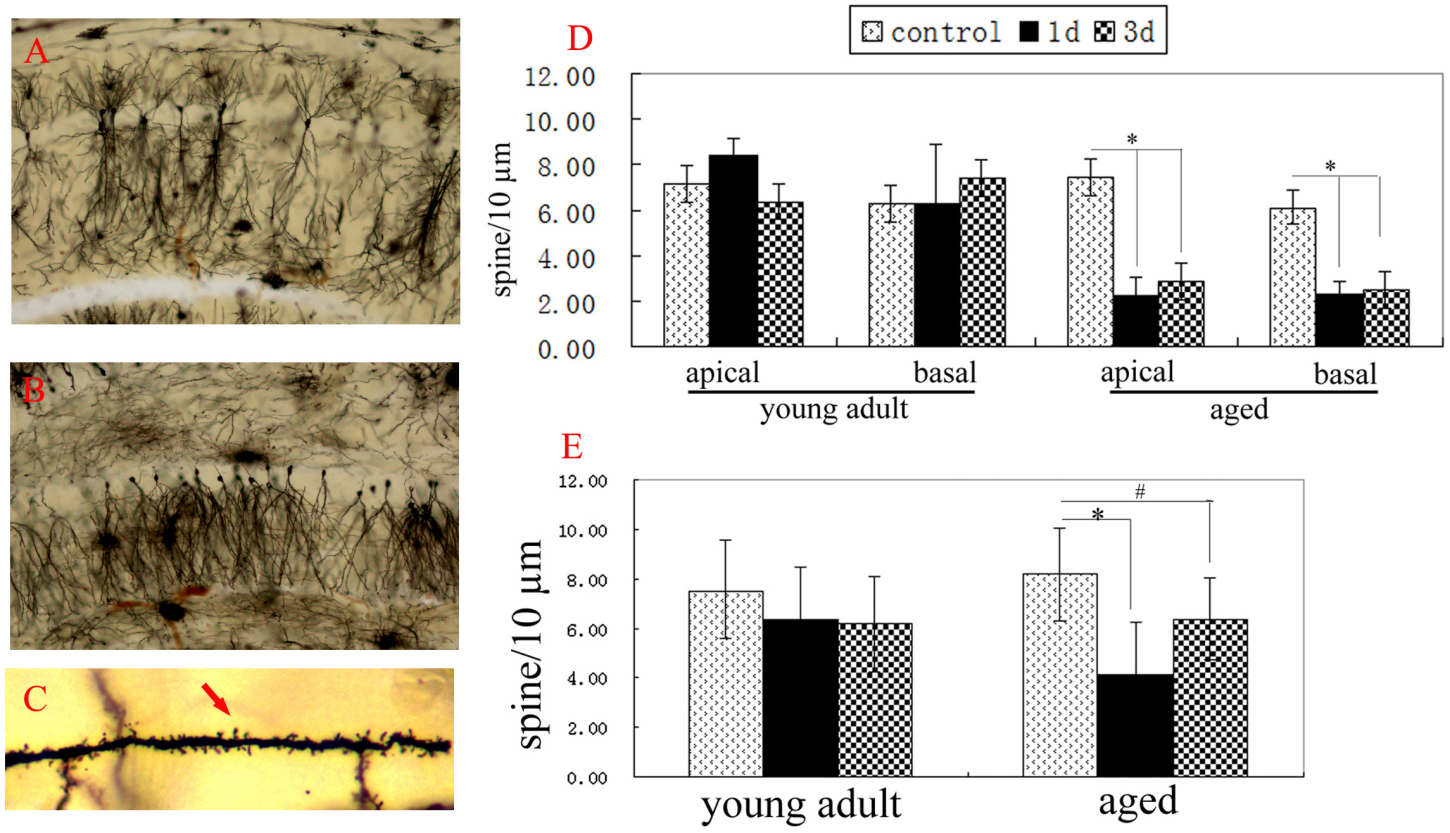
Hepatectomy differentially induced loss of dendritic spines of hippocampal neurons at adult and aged rats. A, neurons of CA1. B, neurons of dentate gyrus (DG). C, showing the dendritic spines (red arrow). D, the spine densities of apical dendrites and basal dendrites of CA1 neurons were analyzed by two-way ANOVA. Compared to the control group, surgery significantly decreased the spine densities of apical dendrites and basal dendrites of CA1 neurons 1d and 3d post-operation (p<0.001, respectively). E, surgery significantly decreased the spine densities of basal dendrites of DG neurons 1d and 3d post-operation (p<0.05, respectively). Data were mean ± SD. *p<0.001 *vs* control. #p<0.05 *vs* control.

**Figure 3:**
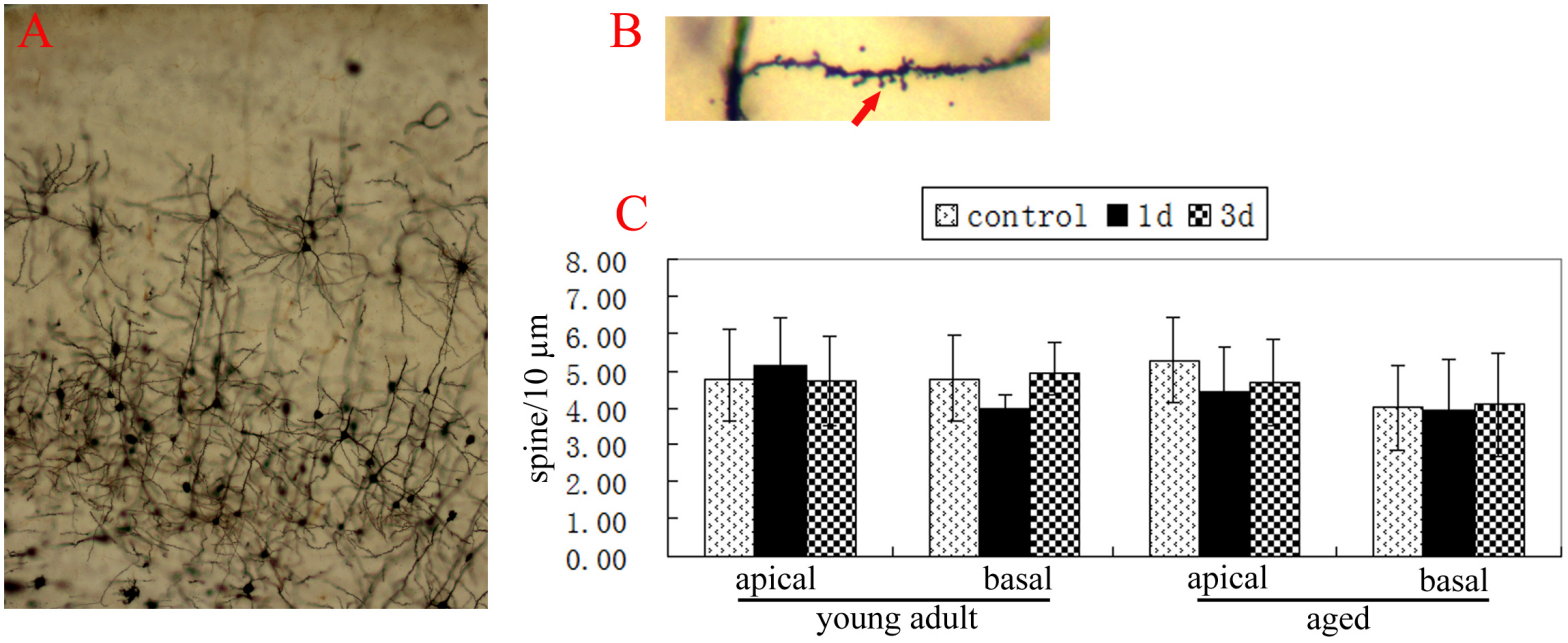
Hepatectomy didn’t decrease spine densities of neuron at cingulate cortex of adult and aged rats. A, neurons of cingulate cortex. B, showing the dendritic spine of neurons at the 2^nd^ layer of cingulate cortex (red arrow). C, the spine densities of apical dendrites and basal dendrites of neurons were analyzed by two-way ANOVA. Compared with the control, no difference was detected 1d and 3d after surgery (p>0.05, respectively). Data were mean ± SD.

Synapse plasticity is the structure base of memory. Changes of dendritic spine density of neurons are usually used to show synapse plasticity [22], [23]. In addition, dendritic spines of neurons are easily affected by the environment [24]. In order to detect the effects of surgery on different brain areas, we detected the changes of dendritic spine densities of neurons at CA1, DG and cingulate cortex of young adult and aged rats after hepatectomy. At normal aged rats, the spine densities of apical dendrites and basal dendrites of neurons at CA1 and cingulate cortex and the spine densities of basal dendrites of neurons at DG all were similar to that of normal young adult rats, respectively (p>0.05) (Fig. 2, 3). Two-way ANOVA analysis showed that spine densities of apical dendrites and basal dendrites of neurons at CA1 and DG of aged rats were affected by surgery (p<0.001 for CA1 apical, CA1 basal, and DG basal, respectively), age (p<0.001 for CA1 apical and CA1 basal; P = 0.405 for DG basal), and the interaction of surgery×age (p<0.001 for CA1 apical and CA1 basal; P = 0. 078 for DG basal). Compared to that of normal aged rats, the spine densities of apical dendrites (2. 26±0.73 for 1d; 2. 86±0.95 for 3d) and basal dendrites (2. 28±0.60 for 1d; 2. 49±0.80 for 3d) of neurons at CA1 of aged rats significantly decreased 1d and 3d after hepatectomy (P<0.001, respectively) (Fig. 2D). The spine density of basal dendrites of neurons at DG of aged rats also obviously decreased 1d and 3d after hepatectomy (P<0.001 for 1d; P = 0.004 for 3d) (Fig. 2E). In contrast, the spine densities of apical dendrites and basal dendrites of neurons at cingulate cortex of aged rats weren’t altered 1d and 3d after hepatectomy, compared to the normal aged rats (P>0.05) (Fig. 3C). These data showed that surgery decreased the spine densities of neurons at CA1 and DG, but not at cingulate cortex of aged rats.

At young adult rats, the spine densities of apical dendrites and basal dendrites of neurons at CA1 and cingulate cortex and the spine densities of basal dendrites of neurons at DG all weren’t altered 1d and 3d after hepatectomy, similar to that of normal young adult rats (p>0.05, respectively) (Fig. 2, 3).

### Hepatectomy differentially induced strong neuroinflammation at hippocampus of aged and young adult rats

**Figure 4:**
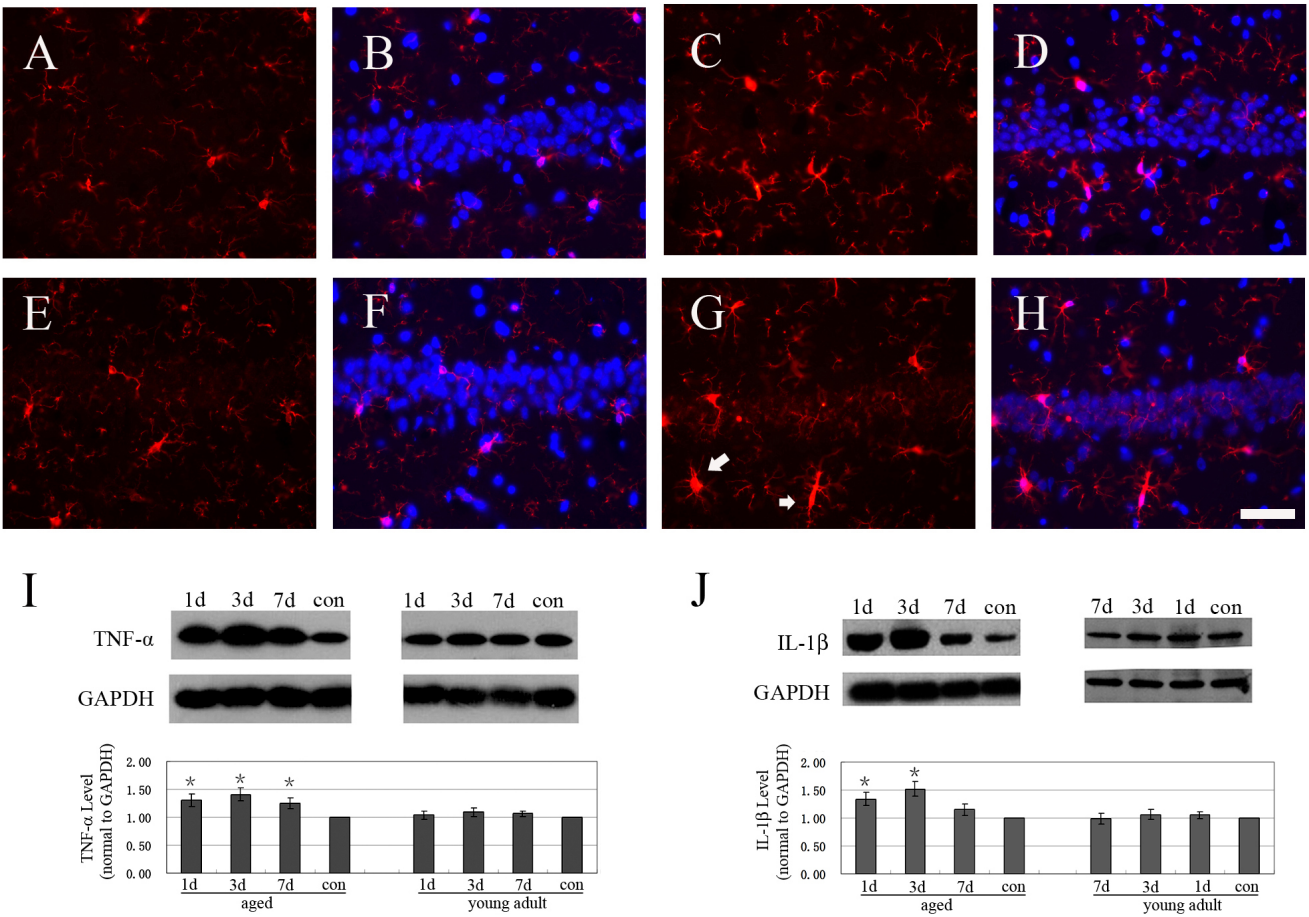
Hepatectomy differentially induced strong neuroinflammation at hippocampus of aged and adult rats. A: Iba1 staining (red) of CA1 at normal young adult rat. B: the merged panel of Iba1 staining (red) and Dapi (blue) of CA1 at normal young adult rat. C: Iba1 staining (red) of CA1 at young adult rat 1d after surgery. D: the merged panel of Iba1 staining (red) and Dapi (blue) of CA1 at young adult rat 1d after surgery. E: Iba1 staining (red) of CA1 at normal aged rats. F: the merged panel of Iba1 staining (red) and Dapi (blue) of CA1 at normal aged rats. G: Iba1 staining (red) of CA1 at aged rat 1d after surgery. Activated microglia (white arrow) was observed. H: the merged panel of Iba1 staining (red) and Dapi (blue) of CA1 at aged rat 1d after surgery. I: western blot of TNF-α at hippocampus. J: western blot of IL-1β at hippocampus. Data were mean ± SD. *p<0.05 vs control. Bar = 50 um.

Inflammation modulates the synapse plasticity of brains at physical and pathological conditions [25]. Previous studies showed that surgery increased the inflammation at the hippocampus of aged rats [12], [26]. Treatment of anti-inflammation drug and blocking the signals of TNF-α and IL-1β all effectively decreased the cognitive impairment induced by surgery [8], [14], [27]. These showed that TNF-α, IL-1β and neuroinflammation played important roles in POCD. Thus we detected the activation of microglia and the expressions of TNF-α and IL-1β in the hippocampus. The size of cell body of microglia increased and rod-like microglia was also observed in aged rats 1d after surgery, compared to the normal aged rats (Fig. 4E–H). No obvious change of microglia was observed at young adult rats 1d after surgery, compared to that of normal control (Fig. 4A–D). Western blot showed that levels of TNF-α and IL-1β at hippocampus of aged rats significantly increased 1d, 3d and 7d after hepatectomy, compared to that of control aged rats (P<0.05) (Fig. 4I, J). In contrast, the levels of TNF-α and IL-1β at hippocampus of young adult rats after surgery were similar to that of control (P>0.05) (Fig. 4I, J).

## Discussion

The aim of the study was to detect (1) whether POCD in animals involves other brain areas besides hippocampus; (2) how age influences POCD of young adult and aged animals. We found that corresponding to memory impairment of aged rats after surgery, surgery induced obvious loss of dendritic spines of neurons at CA1 and DG of aged rats, but not affected the density of dendritic spine of neurons at cingulate cortex. Surgery didn’t significantly decrease spine density of neuronal dendrites and memory of young adult rats. In addition, surgery induced strong neuroinflammation at hippocampus of aged rats, but not at the young adult rats. These data suggested that surgery differentially induced the loss of neuronal dendritic spine and neuroinflammation at hippocampus of young adult and aged rats, which was the possible basis for postoperative memory impairment of aged rats. These data also suggested that hippocampus was the main target involved in POCD.

Many brain areas including frontal, parietal, temporal, occipital, hippocampal, insular, cingulated, thalamic and cerebellar regions are affected in aged patients with POCD [4]. These surgery-affected brain areas are closely related with memory, information processing, concentration and mental flexibility [4]. However, in the animal studies of POCD, most of them focused on the structure and function of hippocampus [4]. To date, it remained unclear whether surgery affected other brain areas besides hippocampus in animals with POCD. Dendritic spines of neurons were easily affected by the environment [24], and were closely associated with memory [22], [23]. Thus, in the study, we used the changes of dendritic spines of neurons to evaluate the effects of surgery on neurons of hippocampus and cingulate cortex, two very important brain areas involved in POCD of patients. Our data showed that surgery induced obvious memory impairment of aged rats 1d and 3d after hepatectomy, but not at young adult rats. Corresponding to the postoperative memory impairment of aged rats, surgery also obviously reduced the spine densities of dendrites of neurons at CA1 and DG of aged rats 1d and 3d after hepatectomy, but not at cingulate cortex neurons of aged rats and at the neurons of CA1, DG and cingulate cortex of young adult rats. These data showed that (1) the loss of spines of neurons at CA1 and DG was closely associated with postoperative memory impairment of aged rats; (2) hippocampus was the main target impaired by surgery. This was consistent with our previous research of aged patents with surgery [2]. In aged patients, small hippocampal volume could be an independent risk predictor of POCD [2]. In addition, Bloss et al found that dendritic spines of prefrontal cortex neurons of aged rat were remarkably stable to the stress [22]. Thus it was possible that surgery didn’t affect the dendritic spine density of neurons at cingulate cortex of aged rats. The difference of frontal cortex involved POCD between human and rats reported in this study might be due to the species difference. In addition, anesthesia or surgery, like other stress factors, may provide dual effects on neuroprotection and neurotoxicity [4], [11]. Minor stresses (e.g. general anesthetics at low concentration for short time) may provide neuroprotection [4], [11]. Detrimental stresses (e.g. general anesthesia at high concentration for long duration) may provide neurotoxicity [4], [11]. The stress size of anesthesia or surgery was also the possible reason for the difference of frontal cortex involved POCD between human and rats reported in this study.

Age is the main risk factor of POCD [5]. POCD is usually detected in aged patients with surgery, but not in young adult patients [5]. Low cognitive reservation is thought to be the reason for occurrence of POCD at aged patients [2]. However the question is how age influences POCD of young adult and aged animals. Thus we first detected the spine density of neurons at young adult and aged rats after surgery. Our data showed that in contrast to the significant loss of dendritic spines of neurons at CA1 and DG of aged rats after surgery, there were no obvious changes at spine densities of dendrites of neurons at CA1, DG and cingulate cortex of young adult rats. These suggested that surgery induced more obvious impairment of neurons at aged rats than that of young adult rats. Neuroinflammation was closely associated with POCD [8], [14], [27], [28]. So we also detected the activation of microglia and the expressions of TNF-α and IL-1β after surgery. Corresponding to the loss of dendritic spines of hippocampal neurons, microglia was activated and levels of TNF-α and IL-1β were up-regulated at hippocampus of aged rats after surgery. In contrast, activation of microglia and increase of TNF-α and IL-1β were not detected in young adult rats after surgery. These data showed that surgery induced strong neuroinflammation at the hippocampus of aged rats, but not at young adult rats. Similar results were also reported by Cao [26]. They found that surgery induced more durable and stronger inflammation response in aged rats, compared with adult rats [26]. Previous studies showed that intra-hippocampal administration of IL-1β impaired contextual fear memory of rats [29]. Sustained elevation of hippocampal IL-1β levels also produced marked impairments in spatial memory [30]. In addition, over-expressing TNF-α in the brain (TNF-α transgenic mice) impaired leaning of adult mice [31]. Intra-hippocampal administration of TNF-α impaired hippocampal-dependent working memory [32]. Blocking the signals of TNF-α and IL-1β effectively decreased the cognitive function impairment induced by surgery [8], [14], [27]. These information showed that increase of TNF-α and IL-1β was detrimental in learning and memory. Based on the above information, we thought that strong neuroinflammation was possible mechanism for significant loss of dendritic spines of hippocampal neurons of aged rats, finally led to POCD.

Briefly, our data show that partial hepatectomy-induced POCD mainly involves hippocampus impairment and differential loss of neuronal dendritic spines and neuroinflammation at hippocampus are most likely the mechanism for the formation of POCD in aged rats.
